# Strengthening Nepal’s Female Community Health Volunteer network: a qualitative study of experiences at two years

**DOI:** 10.1186/1472-6963-14-473

**Published:** 2014-10-09

**Authors:** Dan Schwarz, Ranju Sharma, Chhitij Bashyal, Ryan Schwarz, Ashma Baruwal, Gregory Karelas, Bibhusan Basnet, Nirajan Khadka, Jesse Brady, Zach Silver, Joia Mukherjee, Jason Andrews, Duncan Smith-Rohrberg Maru

**Affiliations:** Possible, Bayalpata Hospital, Ridikot, Achham, USA; Department of Medicine, Brigham and Women’s Hospital, Boston, MA USA; Department of Medicine, Children’s Hospital of Boston, Boston, MA USA; Medic Mobile, Kathmandu, Nepal; San Francisco, CA USA; Montana State University, Bozeman, MT USA; Division of Global Health Equity, Brigham and Women’s Hospital, Boston, MA USA; Department of Global Health and Social Medicine, Harvard Medical School, Boston, MA USA; Partners In Health, Boston, MA USA; Division of Infectious Diseases and Geographic Medicine, Stanford University School of Medicine, Stanford, CA USA

**Keywords:** International hlth, Public health policy, Developing countr, Health services, MCH

## Abstract

**Background:**

Nepal’s Female Community Health Volunteer (FCHV) program has been described as an exemplary public-sector community health worker program. However, despite its merits, the program still struggles to provide high-quality, accessible services nation-wide. Both in Nepal and globally, best practices for community health worker program implementation are not yet known: there is a dearth of empiric research, and the research that has been done has shown inconsistent results.

**Methods:**

Here we evaluate a pilot program designed to strengthen the Nepali government’s FCHV network. The program was structured with five core components: 1) improve local FCHV leadership; 2) facilitate structured weekly FCHV meetings and 3) weekly FCHV trainings at the village level; 4) implement a monitoring and evaluation system for FCHV patient encounters; and 5) provide financial compensation for FCHV work. Following twenty-four months of program implementation, a retrospective programmatic evaluation was conducted, including qualitative analysis of focus group discussions and semi-structured interviews.

**Results:**

Qualitative data analysis demonstrated that the program was well-received by program participants and community members, and suggests that the five core components of this program were valuable additions to the pre-existing FCHV network. Analysis also revealed key challenges to program implementation including geographic limitations, literacy limitations, and limitations of professional respect from healthcare workers to FCHVs. Descriptive statistics are presented for programmatic process metrics and costs throughout the first twenty four months of implementation.

**Conclusions:**

The five components of this pilot program were well-received as a mechanism for strengthening Nepal’s FCHV program. To our knowledge, this is the first study to present such data, specifically informing programmatic design and management of the FCHV program. Despite limitations in its scope, this study offers tangible steps forward for further research and community health worker program improvement, both within Nepal and globally.

## Background

### Global context: community health workers

Community health workers (CHWs) are key components to public sector health systems in many low- and middle-income countries [1–3]. They perform a variety of functions including educational, preventive, and curative health services [1, 3]. Some CHW programs have shown improved health outcomes within target communities; however, impact has been inconsistent, with other programs providing limited impact or poor service quality [3]. Although consistent oversight, continuing education, monitoring and evaluation, and compensation for work are likely to be key components of effective CHW programs, there remains no consensus on best practices, and empiric data are lacking [1–4]. More broadly speaking, the human-resource and financial-resource constraints of these programs, often run by weakly-resourced public sectors, likely contribute to these variable results, and may be a limiting factor in improving the impact of these programs [3].

### Nepali context: the Female Community Health Volunteer program

The Nepali Ministry of Health and Population (“Ministry”) began its Female Community Health Volunteer (FCHV) program in 1988 [5]. Within this program, FCHVs are recruited from each Village Development Committee (“village”), and are trained to provide community health services with a focus on immunizations, vitamin A supplementation, and maternal-child health [5, 6]. FCHVs, who are selected by pre-established village Mothers’ Groups, serve the wards (within each village) in which they live [5]. Each FCHV serves one ward, or approximately 50 households, and 53% of Nepal’s 48,680 FCHVs have held their posts for over 10 years, with an annual turn-over rate of only 4% [5, 6]. The program has received international attention owing to its high retention rate and its favorable public perception throughout Nepal [7].

Despite its successes, the FCHV program faces challenges: community health services have been inconsistent and of variable quality between districts across the country [8–10]. Previous research has investigated the drivers of FCHV commitment and performance, emphasizing the influences of social respect and moral duty on the low volunteer attrition rate [10]. On the other hand, a lack of financial compensation may impact FCHV effectiveness, with some limited data suggesting the importance of incentives to sustain a regimented work schedule in Nepal [8]. It remains unclear how to improve the consistency and quality of service delivery within the FCHV program, such that all Nepali—including the most socio-economically and geographically marginalized—have access to high-quality community health services.

In this context, we evaluated an innovative pilot program to strengthen the FCHV network within a rural district in western Nepal. This program was assessed at the completion of the first two years of implementation. The goal of this evaluation was to determine whether the five core components of the program were valuable additions to the pre-existing FCHV program, as perceived by the program participants and community members, and to identify programmatic challenges and implementation barriers. The lessons learned from this evaluation have implications for the improvement of the FCHV network in Nepal and for other similar CHW programs globally.

## Methods

### Setting

Since 2009, the Ministry has been working in a formal public-private partnership with non-governmental organization Possible to bolster clinical and community health services at the Ministry’s Bayalpata Hospital, in the remote western district of Achham, Nepal. Achham is the third poorest district in the country, and is home to a primarily agrarian population of 287,000 people who were heavily affected by the recent civil conflict [7]. Infant mortality rates are 65 per 1,000 births, and fewer than 1 in 5 deliveries take place in a health facility [7, 11].

### Programmatic design

In 2010, the Ministry and Possible began a pilot program to strengthen the pre-existing FCHV network in the villages surrounding Bayalpata Hospital. The program was composed of five core components, designed specifically to address known problems reported within the FCHV program throughout the country:

1. *Local FCHV Leadership*: Under the pre-existing Ministry program, FCHVs reported once per month to their local health post. Outside of this, there was limited oversight and support for FCHV work on a daily basis. In order to better facilitate and oversee FCHV work, this program established the role of Community Health Worker Leader (CHWL). CHWLs are literate residents of their villages who were nominated by pre-existing village Mothers’ Groups and hired as salaried staff members of Bayalpata Hospital. Each CHWL oversees an average of 9 FCHVs within their village and is responsible for communicating directly with the staff at the local health posts and at Bayalpata Hospital. CHWLs are overseen by a Director of Community Health (DCH) based at Bayalpata Hospital. This enhanced village-level leadership structure was designed to increase oversight and support for FCHVs while simultaneously improving ties between individual community members and their local health centers.
2. *Weekly Village-level FCHV Meetings*: In the pre-existing FCHV system, FCHVs had met with the health worker at their health post once per month. Given other work demands and time constraints, health post staff members had limited capacity to spend time with them to provide guidance or review patient problems. To address this, CHWLs conduct weekly meetings with their village’s FCHVs, reviewing patient encounters and any challenges involved in their work. CHWLs subsequently review these patient encounters with the DCH during weekly DCH-CHWL meetings, receiving guidance from the DCH and Bayalpata Hospital clinical staff, which they are then able to convey to FCHVs at follow-up meetings.
3. *Weekly Village-level FCHV Trainings:* Over 70% of FCHVs in the pilot program are illiterate with minimal formal education, which limits their health literacy capacity. To address this, the program provides thirty minute weekly trainings for FCHVs on health-related topics relevant to their patients. Weekly DCH-CHWL meetings cover materials that CHWLs then re-teach to FCHVs. Additionally, every two months, FCHVs from multiple villages come together for more intensive day-long trainings.
4. *Weekly Monitoring and Evaluation:* Previously, FCHVs had reported tally-based data of their patient encounters to their health post once per month. This data was often not reviewed and FCVHs rarely, if ever, received feedback about the quality or content of the data. To address this, CHWLs collect FCHV data regarding weekly patient encounters, which are then evaluated by the DCH to monitor trends and provide feedback to CHWLs and FCHVs. At the end of each month, the DCH presents aggregate monthly data to FCHVs, CHWLs, and Bayalpata Hospital staff which, in turn, helps to identify areas for specific patient needs or for more general programmatic improvement.
5. *FCHV Compensation*: Most FCHVs in Achham live in extreme poverty. Because FCHV work is not salaried in the pre-existing Ministry system, competing household, agricultural, and other professional responsibilities pose barriers to consistent, high-quality FCHV work. To overcome these barriers, financial and non-financial incentives are provided to FCHVs through this pilot program. FCHVs receive NR 200 (approximately USD $2.25) for weekly responsibilities (8–12 hours of work per week). Non-financial incentives are provided in the form of weekly meeting lunches, uniforms, health equipment, supplies, and community recognition. CHWLs and the DCH receive salaries from Bayalpata Hospital.

### Study design

We conducted a twenty-four month (September, 2010 – August, 2012) retrospective study of the implementation of this pilot program. We obtained ethical review for this research from the Nepal Health Research Council (Kathmandu, Nepal) and the Partners Healthcare Institutional Review Board (Boston, USA). Focus group discussions (n = 3) and semi-structured interviews (n = 27) were held with involved staff and community members. Two villages’ FCHV’s (n = 18), all CHWL’s (n = 9), and community members from Bayalpata Hospital’s Community Advisory Board (n = 5) were invited to participate in the focus group discussions. These discussions and interviews examined the program’s impact on FCHV supervision and leadership, training, work participation, monitoring and evaluation, and work incentives and satisfaction. Verbal informed consent was received from all participants. No children or patients were involved as study subjects.

A grounded theory approach was used for qualitative data analysis. The content of focus group discussions and interviews were reviewed and coded. Primary categories were identified, which included the effects of the program on leadership, regular work meetings and consistent participation, trainings, monitoring and evaluation, and compensation for work. The results of these analyses are described below. Programmatic process metrics were assessed using weekly monitoring and evaluation data. Descriptive statistics were calculated for programmatic process metrics and cost data.

## Results

### Focus group discussions and interviews

Qualitative data analysis revealed that the program was received positively by focus group participants and interviewees. In the focus group discussions, there was consensus from the participants that the core components of the program were valuable additions to the pre-existing FCHV network. Specific benefits of the program were identified and are summarized, along with selected quotations from focus group discussions and interviews:

**Local Leadership Structure:** Strong local leadership of FCHV work improves consistency and accountability of FCHV work.

DCH: “Yesta saral ebam bistrit rup le byakhya gariyeka ra niyamit suchana aadan pradan ka pranali le jawabdeyita tatha samuhik ekata ra bal ko bridhi ma sahayog puryauchhan. Pahile mahila swasthya swayam sewika haru le aafno gaabisa ka didi bahini haru sanga ahile jattiko sahakarya garna paunu bhayeko thiyena.”

(These well-defined and regular communication pathways enhance accountability and cultivate team-building among the FCHVs, who previously did not interact as much with the other FCHVs in their villages).

FCHV: “(Aafno ghar ko) aru kaam bhaye pani ta jaai nahune ho bhane jaaincha!”

(Even when we have household work to do, we go when they call us for something that we must attend to).

**Weekly Village-level FCHV Meetings:** FCHVs value the opportunity for regular, structured contact.

FCHV: “Yo baithak hapta ko ek choti matrai hoina din dinai ya hapta ko dui tin choti basna mildaina? Kati birami haru lai hami le aaitabar nai herchau, usko barema ek dui din bhitrai khabar puryauna paye ramro hunthyo ki?”

(Couldn’t we meet more often, perhaps twice or thrice a week instead of once a week? We visit a lot of patients on Sunday; it would be great if we could communicate with the hospital regarding the patient within one or two days of the first visit).

**Weekly FCHV trainings:** FCHVs value continued training opportunities, both new lessons and review of previously-taught lessons.

FCHV: “Sarhai ramro kura haru sikaunuhuncha (health post ma) tara hami lai badelgada bata sikaune kura chuttai kisimka hunchan. Badelgada ra hamro yeta (gaun ma) hune baithak ma pahile nasuneka, pahile nasikeka kura sikchau.”

(We learn great things at the health post. However, the topics taught at Bayalpata Hospital and the ones discussed at our weekly meetings are different than what we are taught at the health post. We are taught new things at Bayalpata Hospital).

CHWL: “Taalim dohoryaune kallai ichhya hudaina? Kuda gari ekohoro gaye ta tyo kuda ko matlabai bhayena. Ekohodi kuda gareko jindagi ma ka rahala ta?”

(Repetition is essential. It doesn’t really help if we are taught something just once in life. It’s good to repeat).

**Weekly Monitoring and Evaluation of FCHV Patient Encounters:** FCHVs value the opportunity to get regular feedback and guidance regarding their patient encounters.

FCHV: “Pahile-pahile mahina ma ekchoti report bujhauna gainthyo, didi-bahini bhet hune nahune thaha hunthena, kahile bhetinthyo kahile bhetinthena. Aajkal ta haptai pichhe ekchoti sangai basincha, chalphal garincha, afno kaam ko dukha sukha badincha, naya naulo kura sikincha.”

(Previously, we used to submit reports at the health posts once a month; sometimes we met our fellow FCHVs and other times we did not. Now we all sit together and discuss our work at least once a week and learn new things).

**Compensation for FCHVs:** Financial and non-financial compensation, including community recognition, are important motivators for FCHV work, especially in the setting of competing domestic and professional demands.

FCHV: “Kaam dherai chha tara subidha kehi chhaina..chora-chori chhan, khuwaunai padyo.”

(We have to work a lot without any benefits… We have children; we need to feed them).

FCHV: “Pahile kohi sarhai birami pareka, marnai lageka bhaye matrai bhetna jaainthyo, aajkal sabai ko sancho bisancho bela bela bheterai shodhincha, kallai ke kasto chha thaha bhairakcha.”

(Previously, we used to only visit critically ill patients when called upon. Now, we regularly monitor the health status of our community members).

FCHV: “Gaun ma birami parda sab bhanda pahile hamilai bolauchhan. Aafna chimeki ra samudaya ko herchah garna paunu hamro lagi garba ko kura ho.”

(Whenever someone is ill in our community, they come looking for us first. It’s a matter of pride for us to be taking care of our neighbors and community members).

### Programmatic challenges

While the program was received positively, three specific challenges to successful program implementation were identified through focus group discussions and interviews:

- ➢ *Illiteracy*: The limited literacy of the majority of FCHVs constrained the extent of detailed data collection for monitoring and evaluation. CHWLs are all literate, but by themselves, were not capable of logging all individual encounters, nor verifying each individual encounter, thus creating a reliance upon FCHVs’ memory and/or their assistance by literate family members/colleagues.
- ➢ *Professional respect*: Historically, there has been a lack of professional respect for FCHVs by some of the more highly-trained healthcare workers at health posts and hospitals. In tandem with the novel CHWL position (which was previously unknown to other healthcare workers), this disrespect posed barriers of professional legitimacy, and occasionally led to an unwillingness to work with the FCHVs and CHWLs by the healthcare workers.
- ➢ *Geographic limitations*: The long distances between some of the villages and Bayalpata Hospital (some as far as four hours walk each way) limited the ability of FCHVs to consistently attend group trainings, and their ability to effectively refer/transport patients from their villages to the hospital when the health posts’ services were insufficient.

### Programmatic data

**Figure 1:**
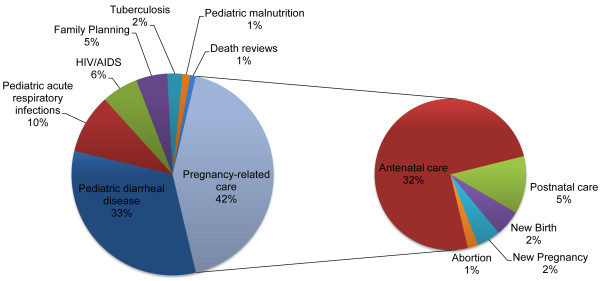
**Distribution of FCHV patient encounters** ***.*** *Pregnancy-related care (42%), pediatric diarrheal diseases (33%), and pediatric acute respiratory infections (10%), were the three most common encounter types.*

During the study period, the pilot program expanded from three to nine villages. At the completion of the study period, the program included 92 FCHVs, nine CHWLs, one DCH, two Assistant DCHs, and a catchment area of 20,905 people. Throughout the study period, weekly village-level CHWL-FCHV meetings and trainings maintained high attendance rates: aggregate average weekly attendance for all villages was 99%. FCHVs conducted an average of 183 patient encounters per village per month, with pregnancy-related care (42%), pediatric diarrheal disease (33%), and pediatric acute respiratory infections (10%) being the three most common encounter types (Figure 1).

### Programmatic costs

Total program costs during the study period were NR 2,709,995 (USD $30,111). Financial compensation accounted for 95% of total costs, with 45% for FCHVs, 19% for CHWLs, and 31% for the DCH and Assistant DCHs. The remaining 5% was administrative costs. Based on monthly expenditures in the program’s 24th month, costs are approximately NR 154 (USD $1.72) per patient served per annum.

## Discussion

The Nepali FCHV program has been internationally acclaimed as a model CHW program, but within Nepal, has been inconsistent in its ability to deliver high-quality services on a national scale. We have presented qualitative data from an innovative pilot program to strengthen Nepal’s FCHV network in one district of the country. The data from this evaluation demonstrate potential value for the five core program components, suggesting that these components may be important for improving Nepal’s FCHV program. To our knowledge this is the first study to present such data, which may inform further programmatic design for the FCHV program.

### Study limitations

The data presented in this study may inform potential improvements to the FCHV program, as reported by program participants and community members. However, this study was conducted in a retrospective manner, in a single district in Nepal, within a single socio-cultural and economic context. These factors limit its external validity on a national level throughout Nepal and also on an international level as far as other similar CHW programs are concerned. Additionally, given the lack of health outcomes data associated with this study, it is not possible to assess whether these programmatic improvements would lead to changes in population-level health indices.

## Conclusions

This study provides further insights into FCHV programmatic design and implementation which may improve community-based service provision, but is limited in its scope and impact assessment. To further clarify best practices within Nepal, or globally in other similar CHW programs, additional operational and outcomes research is needed. Testing the program presented in this article regionally within western Nepal in a large-scale effectiveness trial [12] will help to define its impact and identify areas for potential improvement in the program’s model. Similar implementation research trials in Nepal and globally will help to clarify best practices and, in turn, to maximize the effectiveness of CHWs within the villages they serve.

## Abbreviations

FCHV: Female Community Health Volunteer
CHW: Community health worker
CHWL: Community Health Worker Leader
DCH: Director of Community Health.
